# Prognostic Value Estimation of Monoamines Systemic Level in Retinopathy of Prematurity in Experiment

**DOI:** 10.17691/stm2021.13.3.05

**Published:** 2021-06-28

**Authors:** L.А. Katargina, N.А. Osipova, А.Y. Panova, N.S. Bondarenko, Yu.О. Nikishina, А.R. Murtazina, М.V. Ugrumov

**Affiliations:** Professor, Deputy Director for Science, Head of the Department of Children Eye Pathology; Helmholtz National Medical Research Centre of Eye Diseases, 14/19 Sadovaya-Chernogryazskaya St., Moscow, 105062, Russia; Researcher, Department of Children Eye Pathology; Helmholtz National Medical Research Centre of Eye Diseases, 14/19 Sadovaya-Chernogryazskaya St., Moscow, 105062, Russia; Junior Researcher, Department of Children Eye Pathology; Helmholtz National Medical Research Centre of Eye Diseases, 14/19 Sadovaya-Chernogryazskaya St., Moscow, 105062, Russia; Researcher, Laboratory of Nervous and Neuroendocrine Regulations; Koltzov Institute of Developmental Biology of Russian Academy of Sciences, 26 Vavilov St., Moscow, 119334, Russia; Researcher, Laboratory of Nervous and Neuroendocrine Regulations; Koltzov Institute of Developmental Biology of Russian Academy of Sciences, 26 Vavilov St., Moscow, 119334, Russia; Junior Researcher, Laboratory of Nervous and Neuroendocrine Regulations; Koltzov Institute of Developmental Biology of Russian Academy of Sciences, 26 Vavilov St., Moscow, 119334, Russia; Professor, Academician of the Russian Academy of Sciences, Head of the Laboratory of Nervous and Neuroendocrine Regulation; Koltzov Institute of Developmental Biology of Russian Academy of Sciences, 26 Vavilov St., Moscow, 119334, Russia

**Keywords:** retinopathy of prematurity, norepinephrine, dopamine, L-DOPA

## Abstract

**The aim of the investigation** was to study a systemic level of L-DOPA, dopamine, and norepinephrine, and assess their prognostic value in retinopathy of prematurity (ROP) development on an experimental disease model.

**Materials and Methods:**

The investigation was carried out on infant Wistar rats (n=36) divided into a study group (rat infants with experimental ROP, n=17) and a control group (n=19). The animals of both groups were sacrificed on days 14, 21–23, and on days 28–30. The choice of the indicated periods corresponded to the key stages of ROP development in an experiment and was based on the findings of our previous histological studies. Dopamine, L-DOPA, and norepinephrine levels in infant rat blood plasma samples were determined.

**Results:**

On day 14 of the experiment (the period corresponds to the pathological neovascularization induction in the applied model and preclinical ROP in children), mean L-DOPA level in infant rats with ROP (0.31 ng/ml) was significantly decreased compared to that in the controls (0.42 ng/ml) (p≤0.01). On days 21–23 of the experiment (the period corresponds to the growth of pathological extraretinal neovascularization in the applied model and ROP stage 3 in children) the systemic level of L-DOPA was still statistically reduced in the study group (0.87 ng/ml) compared to the control group (1.53 ng/ml) (p≤0.01). On days 28–30 of the experiment (the period corresponds to the regress of neovasculature in the applied model and a spontaneous ROP regress stage in children) the L-DOPA level in blood plasma in the study group (0.33 ng/ml) showed an insignificant upward tendency in reference to the controls (0.21 ng/ml). Mean dopamine and norepinephrine levels had no difference in the groups under study of infant rats within all follow-up periods.

**Conclusion:**

Low systemic level of L-DOPA at the preclinical stage of experimental ROP should be considered as a laboratory prognostic criterion of a developing pathological process; it will enable to use the criterion when working out the measures to optimize the existing screening system for the disease in children.

## Introduction

Retinopathy of prematurity (ROP) is a severe vasoproliferative vitreoretinal eye pathology in premature children. It is one of the primary causes of irreversible bilateral impairment of visual functions in children [1].

Impaired retinal angiogenesis underlies ROP clinical presentations, it starts on week 16 of prenatal development and completes by term birth. On ocular fundus of premature children in the norm, there are always found avascular zones on retinal periphery, they extend all the more, the less the child’s gestational age at the moment of examination. The presence of such avascular zones is not a disease: it is the evidence of retinal hypoplasia, its incomplete vascularization, and, therefore, the possibility of ROP development at a later stage. Predictability of the disease onset right at this stage is of great clinical relevance.

ROP course is commonly divided into two phases: active and regressive, or cicatricial. According to the international classification [2], 5 stages of an active ROP phase are distinguished:

stage 1 — there is a thin flat demarcation line of white color, at the boundary of vascular and avascular retina;

stage 2 — the formation of a ridge with volume between vascular and avascular retina (it must be emphasized that in 70–80% cases, in ROP stages 1–2, a spontaneous disease regression can occur with minimum residuals on ocular fundus);

stage 3 is characterized by the manifestation of extraretinal fibrovascular proliferation at the ridge area; if the process is not lengthy (1–2 h circles), a spontaneous regression can occur; however, further disease progression requires medical intervention since continued extraretinal tissue growth results in an irreversible effect and significant vision function loss;

stage 4 — partial retinal detachment;

stage 5 — complete or total retinal detachment.

The gold standard of ROP therapy is laser coagulation of the avascular retinal zone at the threshold stage of the disease. Another treatment option, which has gained currency recently, is anti-VEGF therapy. However, both techniques have certain limitations and insufficient efficiency to treat severe atypical ROP forms occurring in small premature infants. The prevalence of such forms in recent years has increased considerably due to the progress in neonatology. It makes the problem of improving ROP screening and prevention very urgent.

The search for new criteria for ROP prognosis is intimately associated with deep understanding of the disease pathogenesis. Experimental modeling ranks high in studying the mechanisms of ROP development. Retinal neovascularization models are applied extensively on rodents, in particular, infant rats, due to the strong resemblance of experimental ROP in infant rats and the disease in children (pathological development of retinal vessels in animals in dynamics corresponds to avascular zones, pre-retinopathy, and stages 1–3 of an active phase of ROP with subsequent spontaneous regressive disease), as well as a number of advantages when working with small laboratory animals [3, 4].

It should be noted that the study of various pathogenetic factors in intraocular structures in animals is of primarily fundamentally pathogenetic importance. Such studying carries the basis to develop novel approaches to the pathology treatment; however, the assessment of the level of the corresponding factors in children has evident limitations [5, 6]. In this regard, the study of their systemic level appears to be more promising.

**The aim of the investigation** was to study a systemic level of L-DOPA, dopamine, and norepinephrine, and assess their prognostic value in retinopathy of prematurity on an experimental disease model.

## Materials and Methods

The study was carried out on 36 infant Wistar rats according to GOST 53434-2009 “Principles of Good Laboratory Practice”, Russian Federation Chief State Medical Officer resolution No.51 dated August 29, 2014 “Concerning Approval of sanitary regulations 2.2.1.3218-14 “Sanitary and epidemiological requirements for the device, equipment and maintenance of experimental biological clinics (vivariums)”, Federal Law No.61-FZ dated April 12, 2010 “Concerning drug circulation”. The research protocol was approved by the Ethics Committee of Helmholtz National Medical Research Centre of Eye Diseases (Moscow, Russia).

To simulate an experimental ROP, newborn rats (n=17) on day 14 were placed in an incubator with their mothers (the age of female rats was 6–8 months). Every 12 h oxygen concentration in the incubator was changed from 60 to 15%. Then infant rats were placed under normal conditions with normal oxygen concentration (21%). Throughout the experiment, constant temperature (26°С) and light regimes (12 h — day, 12 h — night) were maintained. We developed and described the present experimental ROP model before [7], it being confirmed by immunohistochemical and histological studies revealing the disease characteristics corresponding to stages 1–3 of an active phase of ROP in children.

A control group included newborn rats (n=19), which were kept under the conditions of normal oxygen level (21%) since birth.

The animals of both groups were sacrificed on days 14, 21–23, and 28–30, their heart blood being drawn. The choice of the specified periods is determined by their correspondence to the key stages of ROP in experiment [7].

To determine catecholamines, blood was drawn into a test tube with 5% solution of ethylenediaminetetraacetic acid (EDTA) (Sigma-Aldrich, USA), 30 μl, and 10% solution of sodium metabisulphite (Sigma-Aldrich), 10 μl. Then plasma was separated from formed elements by centrifuging at 1350 g within 10 min, followed by adding 50 pmol of 3,4-dihydroxybenzylamine (DHBA) (Sigma-Aldrich) in 0.1 Н HClO_4_. To release from high molecular weight proteins, the plasma was centrifuged at 16,500 g for 20 min. Before determining catecholamines, the samples were extracted by aluminum oxide deposition. After that, in plasma of each sample, we determined L-DOPA, dopamine, and norepinephrine by high-performance liquid chromatography with electrochemical detection. Separated was performed on a reverse-phase column ReproSil-Pur, ODS-3, 4x100 mm, the pores being 3 μm in diameter (Dr. A. Marsch Ammerbuch-Entringen, Germany) at 30°С, and the mobile phase rate was 1 ml/min, maintained by the LC-20ADsp liquid chromatograph (Shimadzu, Japan) at potential 850 mV.

### Statistical data processing

The findings were statistically processed using a statistics package, Statistica 10.0. The samplings under study were examined for compliance with the normal distribution using Shapiro–Wilk test. A nonparametric Mann–Whitney U-test was used to determine the statistical significance of the obtained data. The differences were significant if p<0.05. The findings were presented as mean value ± standard error (M±SE).

## Results

The specified period — day 14 of the experiment — corresponds to the induction of pathological neovascularization in experimental ROP, according to previous histological examinations we performed [7], and preclinical ROP in children. At this stage, mean L-DOPA in blood plasma of experimental infant rats (0.31 ng/ml) was significantly lower compared to the same parameter (0.42 ng/ml) in control animals (p≤0.01) (see the Figure).

**Figure:**
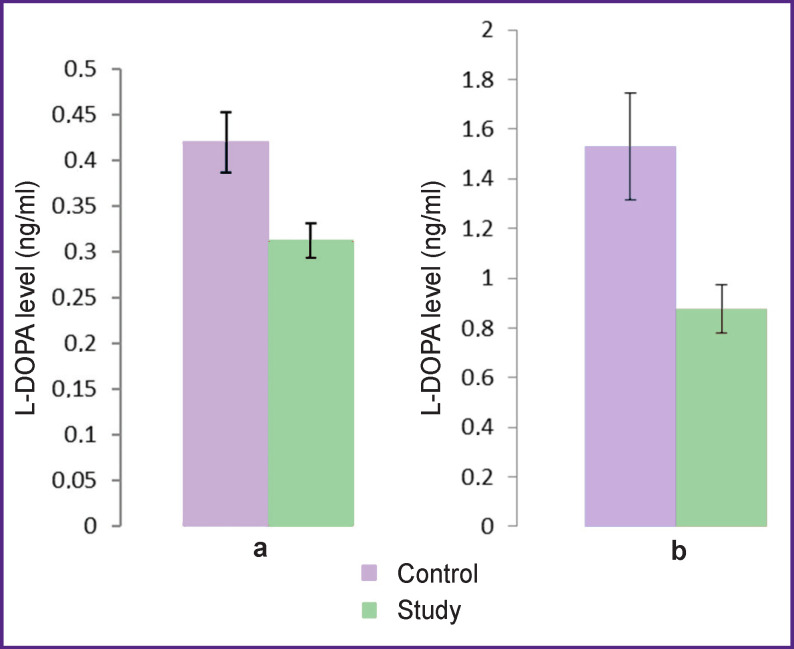
L-DOPA level in blood plasma of infant rats in the study and control groups: (а) on day 14 of the experiment; (b) on days 21–23 of the experiment

On days 21–23 of the experiment (the period corresponds to the active development of pathological extraretinal neovascularization in the applied model [7] and ROP stage 3 in children) systemic level of L-DOPA still was significantly reduced in the study group (0.87 ng/ml) compared to that of the control group (1.53 ng/ml) (p≤0.01), the reduction degree being higher than on day 14.

On days 28–30 of the experiment (the period corresponds to the regress of the newly formed vessels in the applied model [7] and the phase of spontaneous ROP regress in children) its level in blood plasma in the study group (0.33 ng/ml) had just insignificant upward tendency in relation to the controls (0.21 ng/ml).

Mean systemic dopamine level had no significant differences in the study and control groups during experiment stages (Table 1).

**Table 1:** Mean dopamine level in blood plasma of infant rats in the study and control groups within the experiment (ng/ml) (M±SE)

Group	Days		
	14	21–23	28–30
Control	0.04±0.02	0.18±0.02	0.09±0.01
Study	0.03±0.02	0.14±0.02	0.09±0.01

Likewise dopamine, mean norepinephrine in blood plasma of infant rats had no differences in the groups under study within all follow-up periods (Table 2).

**Table 2:** Mean norepinephrine level in blood plasma of infant rats in the study and control groups within the experiment (ng/ml) (M±SE)

Group	Days		
	14	21–23	28–30
Control	3.67±1.73	3.63±1.22	3.42±1.72
Study	3.03±1.54	3.39±1.91	2.82±0.58

## Discussion

The existing ROP screening system involves the ophthalmological examination of all premature babies born at week 35 and weighing under 2000 g at birth [8]. Further monitoring suggests follow-up every other week, once a week, or once every three days depending on a clinical presentation till complete retinal vascularization, spontaneous or induced ROP regress. Numerous examinations produce a stress effect on children, and present a number of possible complications due to the development of oculocardiac and oculopulmonary reflexes, especially in small premature infants [9].

In recent years the attempts are being made to improve the existing protocol of ROP screening. Moreover, two main optimization tendencies are being developed now: telemedicine technologies and the search for supplementary clinical and, particularly, laboratory prognostic criteria, which enable to diagnose children with high ROP risk — to manage them at survival stages, as well as children without or with low ROP risk — to reduce the number of unnecessary examinations [10].

Currently, angiogenic properties of monoamines and their role in the development of vasoproliferative retinal pathology are under study. However, the data on their participation in ROP pathogenesis are so far few and rather contradictory [11–13]. The investigation [6] we carried out recently studied the dopamine content, its precursor — L-DOPA, and norepinephrine in the retina of infant rats with experimental ROP, at different stages of pathology, and the findings indicated L-DOPA and norepinephrine to participate in ROP angiogenesis regulation.

The present investigation aimed at studying the systemic level of monoamines in ROP. It will enable to draw a parallel between the experiment data and the clinical picture, since as it has been mentioned above: the systemic level assessment of different factors is more prospective in immature children compared to the level determination in intraocular structures. Data on L-DOPA are of primary concern since the level of this factor appeared to make significant difference between the animal groups under study at different periods, it indicating a pathogenetic, as well as prognostic role in ROP development.

The obtained data, undoubtedly, enable to consider the low level of this monoamine in blood plasma of infant rats on day 14 of the experiment, i.e. the period corresponding to the onset of pathological retinal angiogenesis or pre-retinopathy in children, as a prognostic feature of extraretinal vasoprolifration in experimental ROP and can serve a kind of a basis to perform clinical trials in order to determine the prognostic value of L-DOPA in blood plasma in immature children as a potential new laboratory criterion for ROP screening.

## Conclusion

Low systemic level of L-DOPA at the preclinical stage of experimental ROP should be considered as a laboratory prognostic criterion of a developing pathological process; it will enable to use the criterion when working out the measures to optimize the existing screening system for the disease in children.
